# Patients’ perspectives about the role of primary healthcare providers in long-term opioid therapy: a qualitative study in Dutch primary care

**DOI:** 10.3399/BJGP.2023.0547

**Published:** 2024-06-25

**Authors:** Lisa Eveline Maria Davies, Elsemiek AW Jansen-Groot Koerkamp, Ellen S Koster, Kelly-Jo Dalusong, Brigitte Koch, Arnt FA Schellekens, Mette Heringa, Marcel L Bouvy

**Affiliations:** Division of Pharmacoepidemiology and Clinical Pharmacology, Utrecht Institute for Pharmaceutical Sciences (UIPS), Utrecht University, Utrecht.; Division of Pharmacoepidemiology and Clinical Pharmacology, UIPS, Utrecht University, Utrecht; SIR Institute for Pharmacy Practice and Policy, Leiden.; Division of Pharmacoepidemiology and Clinical Pharmacology, Utrecht Institute for Pharmaceutical Sciences (UIPS), Utrecht University, Utrecht.; Division of Pharmacoepidemiology and Clinical Pharmacology, Utrecht Institute for Pharmaceutical Sciences (UIPS), Utrecht University, Utrecht.; Division of Pharmacoepidemiology and Clinical Pharmacology, Utrecht Institute for Pharmaceutical Sciences (UIPS), Utrecht University, Utrecht.; Department of Psychiatry, Donders Institute for Brain, Cognition and Behaviour, Radboud University Medical Center; scientific director, Nijmegen Institute for Scientist Practitioners in Addiction, Nijmegen.; SIR Institute for Pharmacy Practice and Policy, Leiden.; Division of Pharmacoepidemiology and Clinical Pharmacology, Utrecht Institute for Pharmaceutical Sciences (UIPS), Utrecht University, Utrecht.

**Keywords:** chronic non-cancer pain, long-term opioid use, opioids, patient perspective, primary care, qualitative research

## Abstract

**Background:**

Over the past decade, long-term use of prescription opioids for chronic non-cancer pain has risen globally despite the associated risks. Most opioid users receive their first prescription in primary care.

**Aim:**

To investigate the perspective of patients who are long-term opioid users in primary care regarding the role of healthcare providers (HCPs) in their prolonged opioid use.

**Design and setting:**

Semi-structured interviews in Dutch primary care.

**Method:**

We recruited patients who were long-term users of opioids for chronic non-cancer pain from seven community pharmacies in the Netherlands. In-depth, semi-structured interviews focused on patients’ experiences with long-term opioid use, access to opioids, and the guidance of their HCPs (primarily their GPs and pharmacists). A directed content analysis was conducted on the transcribed interviews using NVivo.

**Results:**

Participants (*n* = 25) described ways in which HCPs impacted their long-term use of opioids. These encompassed the initiation of treatment, chronic use of opioids, and discontinuation of treatment. Participants stressed the need for risk counselling during initial prescribing, ongoing medication evaluations including tapering conversations, and more support from their HCP during a tapering attempt.

**Conclusion:**

Patients’ perspectives illustrate the important role of HCPs across the spectrum of opioid use — from initiation to tapering. The results of this study underscore the importance of clear risk counselling starting at initial prescribing, repeated medication assessments throughout treatment, addressing tapering at regular intervals, and strong support during tapering. These insights carry significant implications for clinical practice, emphasising the importance of informed and patient-centred care when it comes to opioid use for chronic non-cancer pain management.

## Introduction

Over the past decade there has been a global increase in the use of prescription opioids.^1^^–^^9^ While opioids can provide effective short-term pain relief, long-term use of opioids is associated with various risks, including physical dependence, overdose, and mortality.^3^^,^^6^^,^^10^^,^^11^ Use of opioids for chronic non-cancer pain is generally discouraged, yet around 30% of patients continue to be prescribed opioids long-term.^9^^,^^12^ It is, therefore, important to understand the reasons why patients continue to use opioids long-term.

Previous studies have shown that patients using opioids long-term face challenges while balancing the pros and cons of opioid use amid persistent pain.^13^^,^^14^ Patients’ awareness of risks, coupled with their underestimation of personal risk, pessimism about alternatives, and concerns about withdrawal, contribute to the complexity of long-term opioid use. Despite the crucial role of healthcare providers (HCPs) in supporting these patients, this aspect remains inadequately investigated in current studies.

Primary HCPs play a pivotal role in the treatment of chronic non-cancer pain and in prescribing opioids particularly, as they are at the forefront of patient care.^14^^–^^19^ Management of long-term opioid use is a complex ongoing process, requiring HCPs to constantly balance their wish to alleviate pain with the potential risks of prolonged opioid consumption. However, problematic prescription patterns may arise when patients experience a lack of pain relief after repeated consultations, leaving HCPs unable to explore alternative treatment routes.^18^ Extended waiting times for specialist care may further burden primary HCPs, as they are the first point of contact for patients with chronic pain, highlighting challenges exacerbated by time constraints during patient visits and limited chronic pain management training.^17^^,^^20^

In the Netherlands, GPs primarily prescribe opioids, including tramadol, oxycodone, morphine, and fentanyl, while codeine is discouraged because of limited effectiveness and side effects.^21^^,^^22^ Concerns over rising opioid prescriptions, particularly oxycodone, led to revisions to guidelines in 2021, advocating a more conservative approach to chronic pain management.^23^ HCPs are advised to educate patients about the benefits and risks of opioids, discuss tapering options, engage in shared decision making for goal setting, and address withdrawal and pain management strategies.^24^ In addition, community pharmacists are expected to play a proactive role in counselling patients, monitoring use, and collaborating with GPs on prescribing agreements and individual treatment plans for escalating opioid use.

**Table:** How this fits in

Previous research has shown the pivotal role of primary healthcare providers in managing long-term opioid use for patients with chronic non-cancer pain. This study adds the patient’s perspective, underscoring the importance of improved communication, medication management, regular assessments, and a patient-centred approach, especially during opioid tapering. Clinicians should prioritise these aspects to enhance patient care and outcomes for patients in chronic non-cancer pain management.

It is not known how patients perceive their primary HCPs and how the guidance of HCPs influences their opioid use. Gaining insight into these aspects may help provide improvements to patient care and develop appropriate pain management strategies for the complexities of long-term opioid use. Therefore, this study aims to investigate the patient’s perspective about the role of primary HCPs in their long-term opioid treatment.

## Method

### Study design

A qualitative study was conducted in primary care using in-depth, semi-structured interviews with patients who have been using opioids long-term for non-cancer pain.

### Setting and participants

Patients were recruited between February and June 2021 from seven community pharmacies located in different parts of the Netherlands. Pharmacies were affiliated with the Utrecht Pharmacy Practice network for Education and Research.^25^ Eligibility criteria included being a Dutch-speaking resident, aged ≥18 years, with an opioid indication for chronic non-cancer pain, and having received at least two prescriptions for opioids in the past 7 months with a total supply for at least 3 months, regardless of potency.^26^

The pharmacist contacted eligible patients to ask whether they would like to participate in the study. If interested, patients received a patient information letter. The pharmacist shared patients’ contact details with the research team after patients had given their consent. Thereafter, researchers contacted interested patients via telephone to set an appointment for the interview. Data saturation, defined as the point where further data collection ceases to provide additional thematic insights and codes, was anticipated to be achieved after conducting 15 to 25 interviews.^27^ Parallel analysis was conducted alongside the interviews, and, if data saturation was reached, three additional interviews were scheduled.

### Data collection

An interview guide with open-ended questions, focusing on topics in the prescribing process, was reviewed and refined by the research team. The following key questions were presented to the patient: ‘How did your first opioid prescription conversation take place?’ and ‘Has your GP or pharmacist ever discussed your long-term opioid use?’ Thereafter, corresponding sub-questions were asked to go deeper into specific topics. The interview guide was iteratively refined after four interviews (see Supplementary Information S1 for the full interview guide). Oral informed consent was obtained from all study participants before the start of the interview.

A team of four female researchers (one PhD candidate and one PhD candidate/pharmacist who were both experienced in qualitative research, and two master’s students in pharmacy who had received training in patient communication education) conducted interviews in pairs or triads (referring to the interviewers) via telephone or video call, according to patient preferences. In each interview, one person led the interview, while other researchers observed and addressed any missed questions at the end. Because of COVID-19 restrictions, no interviews were held face-to-face. All interviews were audio-recorded and field notes were made. The researchers had no personal interest in the subject, apart from the fact that this is the subject of their PhD and master’s thesis. No relationship was established with participants before the study. After the interviews, the following patient characteristics were extracted from the patient’s medical record: opioids used during the last 3 months (name, dosage, and units), and the use of opioids in the past year (medication name only). All opioid prescriptions used during the 3-month period were converted into oral morphine equivalent using published conversion factors and divided by 90 days.^28^

### Data analysis

Interview audio-recordings and field notes were transcribed verbatim. Transcripts were not returned to participants for comment. All collected data were anonymised to protect the privacy and confidentiality of the individuals and places involved. All audio-recordings were deleted after final analysis of the data. Data were analysed using directed content analysis with a predefined coding tree based on the processes in pharmacotherapy: initiation of treatment, chronic use of medication, and discontinuation of treatment. This method was chosen to capture patient experiences related to HCP steps, offering a structured and systematic approach, although codes for actual experiences were not predefined.^29^ Four researchers coded all transcripts independently. Coding discrepancies were discussed and reconciled between three researchers. Agreements were made about how to interpret, sort, summarise, and shorten quotes that support a code. Codes were adjusted, added, or removed as needed. Two researchers conducted the initial categorisation of the codes. Concept categories and themes were evaluated with the research team. The final coding tree (see Supplementary Box S1 for details) was applied to all transcripts. NVivo (version 12) was used for data management and analysis. The findings were reported according to the consolidated criteria for reporting qualitative research.^30^

## Results

### Variable

A total of 25 participants were interviewed; seven participants were interviewed via video call (28%) while 18 (72%) were interviewed by telephone. Interviews lasted approximately 25 minutes to 1 hour each. Data saturation occurred after 20 interviews. However, with five more interviews already scheduled across three pharmacies, it was decided to proceed with all five instead of the initially planned three. On average, participants were aged 61 years and most were female (64%) (Table 1). Nearly all participants used opioids for at least 1 year, with oxycodone being the most frequently used opioid as either monotherapy (44%) or in combination with another opioid (24%). See Supplementary Table S1 for a detailed description of participants.

**Table 1.** Participant characteristics, *N* = 25

**Variable**	***n* (%)^a^**
**Female**	16 (64)

**Age, years, mean (SD)**	61 (±10)

**Reason for initial opioid prescription**	
Post-surgical and post-traumatic pain	8 (32)
Musculoskeletal pain	12 (48)
Neuropathic pain	3 (12)
Other pain	2 (8)

**Duration of opioid use**	
<1 year	3 (12)
1–5 years	8 (32)
>5 years	14 (56)

**Average daily OME, mg, median (IQR)**	33.8 (15.8–97.0)

**Used opioids in past 3 months**	
Tramadol	7 (28)
Oxycodone	11 (44)
Morphine	1 (4)
Combination (for example, oxycodone + fentanyl)	6 (24)

a *Unless otherwise stated. IQR = interquartile range. OME = oral morphine equivalent: calculated based on last 3 months of prescriptions. SD = standard deviation.*

**Figure 1.**
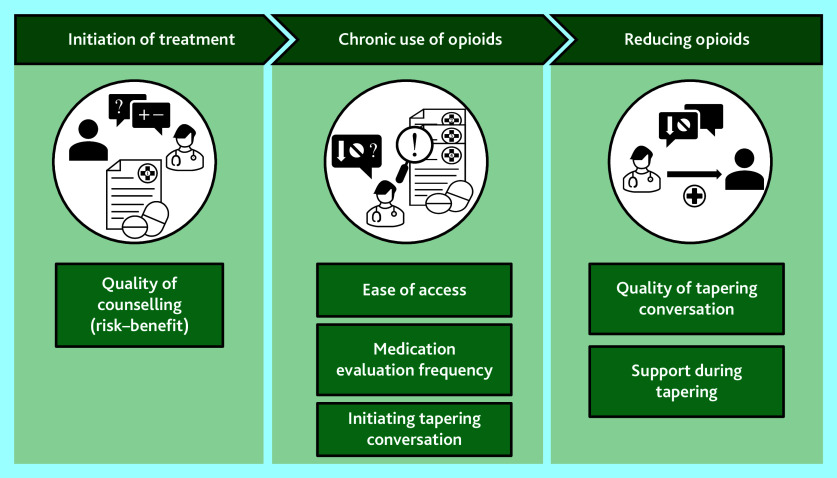
Patients’ perspectives about the role of the healthcare professional in long-term opioid treatment.

The reasons that participants started opioid treatment was because of persistent pain complaints (*n* = 17, 68%), including musculoskeletal, neuropathic, or unspecified pain (such as fibromyalgia), or post-surgical or post-traumatic pain (*n* = 8, 32%) (Table 1). On average, 33.8 mg (interquartile range 15.8–97.0) of daily oral morphine equivalents was prescribed. Nearly all participants reported having a positive relationship with their GP. We identified three themes in primary care: the role of the HCP in initiation of opioid treatment, the role of the HCP in repeat prescriptions, and the role of the HCP in opioid tapering (Figure 1). The main themes are discussed below.

### Initiation of treatment

#### Quality of counselling at first prescription

At the beginning of their treatment, most participants were unfamiliar with opioids. While nearly all participants could remember receiving practical information about how to take the medication and a leaflet, many could not recall specific details about the risks associated with opioids:

*‘The doctors prescribed that* [opioids] *and said: “This will make you sleep well”, and I did. There was no further explanation … it’s prescribed and the doctor said: “If there are any side effects, let me know.”’*

(P24, female, aged 56 years)

*‘They also mentioned some things about it, but I don’t remember exactly. They tell you about it, and I did read the patient information leaflet, but then you don’t really think about it any more. I know what it is, and I know that you can’t just take it across the border without proper authorisation.’*

(P4, female, aged 63 years)

Participants placed significant trust in their HCPs, often disregarding supplementary information (such as leaflets) about risks if not underlined by their HCP. In hindsight, roughly half of participants stated that they had received insufficient counselling regarding the risks from their HCPs:

*‘When I received the opioids there was a package leaflet … but I never read those as it is useless … I go to the doctor for my back pain, and I get medicines and I take those. I could read everything, but … I trust the doctor and the pharmacy.’*

(P17, male, aged 60 years)

Only a few participants mentioned receiving a clear risk–benefit explanation from HCPs, which also included the potential risks of opioid addiction:

*‘When I received a prescription from the GP, they told me that there was a possible risk of addiction and that I had to be careful with my use, which I still am to this day. I only use opioids when I’m in pain. I don’t use it every day.’*

(P7, female, aged 67 years)

In addition, a few participants who did not receive adequate counselling reported becoming aware of opioid risks by reading package leaflets, conducting online research, or learning from news sources, family, or friends. For instance, one participant stated:

*‘I was informed that morphine is a stronger form of pain relief, stronger than paracetamol or ibuprofen. For additional information, I read the package leaflet and looked up information online.’*

(P14, male, aged 68 years)

### Chronic use of opioids

#### Ease of access to opioids

Participants described easily obtaining repeat prescriptions from multiple prescribers with a single phone call. They reported a lack of follow-up questions or comments from their GP or pharmacist, with about two-thirds experiencing no discussion about their repeat prescriptions during appointments:

*‘I only had to call them* [HCP]*, and they will throw it* [opioids] *through my letterbox.’*

(P19, male, aged 46 years)

*‘I receive ninety units. So, I always have enough at home … I made sure that if I had only two boxes left … that I would already order, so I never actually ran out.’*

(P9, female, aged 43 years)

However, some participants mentioned that their HCPs stressed the need to have a conversation before repeating the prescription:

*‘I only need to call, and they will write the prescription for me. Every three months, I have to visit the GP. He asks how things are going, and I always get just thirty* [pills]*.’*

(P25, male, aged 60 years)

On the other hand, there were those who reported that their HCPs seemed hesitant or displeased when asked for a prescription refill, leading them to avoid regular visits to the HCP. Nonetheless, all participants were eventually able to have their prescriptions refilled:

*‘I do not visit the GP regularly to be honest. Cause they do not really cooperate … they do not like the oxycodone and want to stay in control of everything.’*

(P18, female, aged 47 years)

#### Medication evaluation frequency

Most participants stated that their GP had never initiated a discussion about their chronic use of opioids and the potential risks thereof. In hindsight, some patients realised that this gave them a false sense of safety, resulting in them never evaluating the need for opioid treatment:

*‘The last time I had a consultation at the doctor was about nine months ago. That’s not good. There is no guidance at all! … They should have told me that it can be addictive. It is advisable to have an evaluation every two months, as the doctor can’t always keep an eye on it. That way you share the responsibility. However, that didn’t happen.’*

(P19, male, aged 46 years)

Participants who received regular medication evaluations reported being more aware of the risks of their long-term opioid use:

*‘I believe it is good* [to inform people about the risk of addiction]*, because then people know what they take, are taking, and understand that depending on the amount you take, there will be a time where you cannot be without any more. I can* [go without opioids]*, but there are others* [who cannot]*, so it is good they mention it.’*

(P25, male, aged 60 years)

In addition, most of the participants mentioned that their pharmacist had rarely commented on their prolonged use of opioids and mostly only in case of potential overuse:

*‘I had called the pharmacy about the medications* [opioids]*, and they referred back to the doctors who prescribed it. They do check if I do not order too many. They ask how many I am supposed to have, because I sometimes used too many, and then I would be without.’*

(P8, female, aged 62 years)

Only one participant mentioned receiving comments from the pharmacy that made them re-evaluate their prolonged opioid use:

*‘They make you think. I mean if you prescribe something and no one says something then … but the pharmacy was vigilant and said: “Hey, you’ve been using this for a long time. Do you still need it?” I find that a good thing about the pharmacy.’*

(P24, female, aged 56 years)

#### Initiating tapering conversations

Most participants indicated that they had never discussed the possibility of tapering with their HCP during their long-term opioid use. A few did raise concerns about how long they had been using opioids, but were reassured by their HCPs to continue using them:

*‘I once asked the GP: “How long am I able to use* [opioids]*?”, and they said: “In principle, your whole life”, so then I’m not worried about it.’*

(P4, female, aged 63 years)

### Discontinuation of treatment

#### Quality of tapering conversations

In a few instances, discussions regarding tapering were instigated either by GPs or by participants who were insistent on pursuing tapering measures. Paradoxically, despite these conversations, long-term opioid use often persisted without substantial intervention:

*‘I asked: “Is there a possibility that I will be able to get rid of that oxycodone?” and then the GP said: “Let’s try. Try doing this and that, and if you experience any symptoms, come back and we will see.”’*

(P3, male, aged 72 years)

*‘The GP always asks: “How are you doing? Could you manage with one pill per day?” Then I am very honest about it, and say: “Sometimes, yes, it depends on the day … then I will need two pills.” If I explain it to him then he* [GP] *is fine with it, and we continue the prescription.’*

(P25, male, aged 60 years)

Furthermore, discussions regarding long-term opioid use were considered sensitive. Some participants were confident they could quit opioids, if necessary, while others, because of their chronic pain, saw tapering as impractical, and therefore discouraged tapering conversations. Some also had negative experiences with GPs who were critical of opioids, leading them to avoid discussing their use and tapering, with one patient even avoiding GP visits for this reason:

*‘I avoid seeing the GP because he always starts talking about tapering off and how bad it* [the oxycodone] *is. So yeah, then you start avoiding the GP.’*

(P6, female, aged 65 years)

#### Support during tapering

A small group of participants had experience with tapering opioids. Some had attempted tapering without consulting their GP, with some initially succeeding but resuming opioids because of new pain complaints, while others had faced difficulties and lack of support, leading to increased pain and fear of future attempts.

Participants who had attempted to taper their opioid intake under the guidance of their GP also found it challenging to completely reduce their opioid use because of ongoing pain complaints, although no severe cases were reported. While participants acknowledged that their GP could only provide advice and not perform the tapering process for them, some questioned the level of guidance they had received:

*‘I tapered gradually, and it went smoothly … the GP helped me and warned me not to do it alone, so I ensured he was involved.’*

(P3, male, aged 72 years)

One complaint was that participants had to take the initiative to reach out to their HCPs for support and guidance during their tapering attempt:

*‘Well, I had to take the initiative to call them: “It’s not working, the tapering is not working.” I require more guidance on their initiative as well. A more intensive guidance during tapering.’*

(P11, male, aged 57 years)

Furthermore, participants emphasised the importance of their HCPs sharing more detailed information about the steps involved in the tapering process, offering more frequent counselling during the tapering phase, especially concerning the pace of tapering, and providing more shared experiences of successful tapering:

*‘I needed more advice. So, the advice to talk with the GP or pharmacy on how to best taper … but also, more sharing of experience of other people. How to best taper, the consequences and what I might experience … if I know what to expect I think I can persevere more.’*

(P24, female, aged 56 years)

## Discussion

### Summary

Our study provides valuable insights into the perspective of individuals with chronic pain and primary HCPs’ guidance regarding their long-term opioid treatment. Participants highlighted a lack of risk education during the initiation of treatment. Regarding repeat opioid prescriptions, participants emphasised easy access to opioids, inadequate evaluation of medication by HCPs, and a lack of initiation of tapering conversations. When discussions about tapering were initiated, patients often felt restricted and tended to avoid the prescriber. Additionally, when the tapering process began, the guidance provided frequently fell short of meeting their needs.

### Strengths and limitations

This study expands our knowledge about patients’ experiences of opioid use in primary care settings and the impact of HCPs. The study achieved data saturation with a diverse sample from multiple community pharmacies with geographical representation in the Netherlands, and encompassing various opioid medications and dosages.

However, recall bias among long-term opioid users is a notable limitation. Despite this, the study’s significance in grasping broader issues related to opioid use remains. This study lacks data for all candidates approached by pharmacists; feedback indicated that around half of patients approached were willing to participate, potentially introducing bias favouring those open to discussing opioids. The impact is likely minimal, as it does not significantly alter patients’ perspectives of HCP interactions. If any influence exists, those affected are likely to discuss opioid use less than surveyed participants. Users of high-dose opioids may have been less inclined to participate, and our study does not include insights from previous opioid users who had successfully tapered off medication, limiting the range of experience of participants. Additionally, the updated opioid prescribing guidelines issued in November 2021 had had minimal impact as they had not yet been integrated into general practice during the interviews.^23^ Lastly, the study predominantly focused on the patient’s perspective, potentially overlooking the perspectives of professionals and the role that patients themselves play in this process.

### Comparison with existing literature

Previous research found that the duration of opioid use and dose in the first month strongly influence prolonged use.^31^^,^^32^ After 12 days, the risk of long-term use increases to 24%, a figure that rises to 43% after 31 days. Exceeding cumulative doses of 120 oral morphine equivalents within the first month doubles the risk of long-term use. Patients often underestimate their personal risk during chronic opioid therapy, especially when experiencing significant pain, necessitating counselling at the onset of treatment.^14^ Although patients and HCPs find discussing the potential risks of opioids challenging, our findings reveal that patients desire information at the onset about the long-term use in chronic non-cancer pain, and that dose escalation increases dependence risk.^33^ At treatment initiation, patients could also be informed that opioids have comparable effectiveness as other pain relievers such as paracetamol and non-steroidal anti-inflammatory drugs.^34^

Moreover, participants reported ease of access to opioids, often facilitated without direct physician–patient interaction during prescription refills. Despite guidelines advocating regular consultations, a concerning pattern emerged with repetitive opioid prescriptions lacking proper evaluation. This may stem from time constraints, with HCPs assuming patients on chronic opioid therapy are doing well unless they express concerns.^17^^,^^35^ However, over time, the benefits of opioids may diminish, while hidden harms persist. Recognising these issues and responding to patients’ consistent need for more regular consultation underscores the importance of allocating additional time to address concerns, evaluate patients’ conditions, and explore alternatives to opioids. Ensuring that patients can make informed decisions fosters shared responsibility, so reducing potential harm.^36^

Participants noted a lack of tapering conversations and personalised guidance concerning their long-term opioid use, possibly as a result of recognised barriers for HCPs, such as time constraints, limited resources, inadequate training, emotional complexities, trust issues, fear of harming the patient–provider relationship, and limited access to non-opioid treatments.^18^^,^^24^^,^^37^^–^^41^ Interestingly, the patients’ needs align with HCP-proposed strategies to facilitate opioid tapering, emphasising intrinsic patient motivation and tailored tapering plans, incorporating motivational interviewing, timing, and pace adjustments, and counselling about potential pain and withdrawal symptoms during dose reduction.^40^^–^^42^ Conversely, some patients disengaged during negative discussions about long-term opioid use. To mitigate this, HCPs advise acknowledging chronic pain experiences, expressing empathy, and linking pain concerns with safety.^40^^,^^41^ Sensitivity and understanding are crucial when approaching discussions about opioid tapering.

Creating a supportive environment with open communication and empathy is crucial for successful tapering, ensuring patients’ feelings of being heard and understood, and intrinsically motivating them, supports success from both perspectives, benefitting both the patient and the HCP. To address time constraints and waiting lists for pain specialists, a more prominent role for pharmacists or nurses could be beneficial. Recent initiatives, such as a pharmacist-assisted programme, have demonstrated promising outcomes in the management of chronic non-cancer pain and the reduction of opioid prescribing. This intervention encompassed patient notifications, proactive outreach by pharmacists, and the establishment of a patient registry with regularly updated clinical data.^43^ Additionally, a nurse-led telephone follow-up intervention for titrating or tapering opioids showed positive results.^44^

### Implications for research and practice

Our results emphasise the urgency of better communication between HCPs and patients about the risks associated with opioid therapy right from the initial prescription, and the importance of maintaining these conversations throughout subsequent prescriptions. There is a need for improved medication management and regular patient assessments. We advocate for a shift from a purely pain-focused approach to one that considers the patient’s perspective, ensuring a balanced assessment of risks and benefits. Such a transformation requires routine evaluations of pain levels and opioid effectiveness, with close monitoring by both GPs and pharmacists. Additionally, our study promotes a more compassionate and supportive approach to opioid tapering, acknowledging the challenges and potential stigma patients may face during this process. The healthcare system should extend empathy and guidance to support patients effectively throughout their tapering journey.

Future research should focus on developing conversation tools to support patients with chronic non-cancer pain, aligning treatment plans with patient perspectives, and promoting safe opioid use in long-term pain management. Additionally, exploring solutions to reduce HCP burdens could enhance chronic non-cancer pain management while minimising opioid prescribing.

## Data

Because of the confidentiality agreements and the sensitivity of the topic for research participants, supporting data are not publicly available. However, data are available on reasonable request to the corresponding author. It should be noted that raw data are only available in Dutch.
